# 10^−21^-Level optical frequency dissemination over 2067 km of noise-loaded field-deployed fiber network

**DOI:** 10.1038/s41377-026-02299-1

**Published:** 2026-06-22

**Authors:** Fa-Xi Chen, Li-Bo Li, Jiu-Peng Chen, Kan Zhao, Jian-Yu Guan, Yang Xu, Lei Hou, Fei Zhou, Cheng-Zhi Peng, Qiang Zhang, Hai-Feng Jiang, Jian-Wei Pan

**Affiliations:** 1 Hefei National Laboratory, University of Science and Technology of China, Hefei, China; 2 Jinan Institute of Quantum Technology and CAS Center for Excellence in Quantum Information and Quantum Physics, University of Science and Technology of China, Jinan, China; 3 Hefei National Research Center for Physical Sciences at the Microscale and School of Physical Sciences, University of Science and Technology of China, Hefei, China

**Keywords:** Optical metrology, Fibre optics and optical communications

## Abstract

Achieving ultra-stable optical frequency dissemination over long-haul fiber networks is essential for numerous applications. Although optical frequency transfer systems based on optical phase-locked loops (OPLLs) have achieved unprecedented stability levels, their performance is limited by continuous compensation bias caused by noise asymmetry from bidirectional frequency shifts and loss of lock in long-distance, high-noise links. Here, we propose a bias-free noise compensation method based on digital radio-frequency phase recording using a time-to-digital converter, which eliminates residual errors and offers a theoretically unlimited dynamic range for enhanced reliability. By incorporating multifunctional relay stations and hertz-level optical bandpass filtering to improve OPLL robustness, our scalable architecture achieves a frequency instability of $2.9\times {10}^{-21}$ at 1 day over a 2067 km field fiber link under extreme noise conditions (5000 ${\mathrm{rad}}^{2}\,{\mathrm{Hz}}^{-1}\,{\mathrm{km}}^{-1}$ at 1 Hz). Bias correction improves the instability by threefold and breaks through the theoretical limit of uncalibrated systems. The setup maintains continuous phase lock for over four days, and noise purification enables virtually unlimited link extension. This advance establishes a robust, field-deployable optical frequency network compatible with standard telecommunication infrastructure.

## Abstract (web summary)

Bias-free digital TDC phase recording and cascaded relay stations with hertz-level purification achieve 2.9 × 10^−21^ instability over 2067 km noisy field fiber, breaking conventional limits

## Graphical abstract

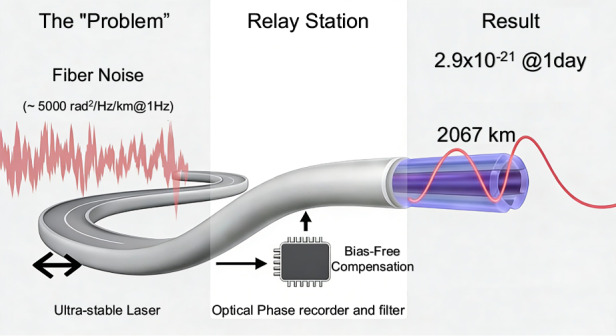

## Introduction

The global dissemination of ultra-stable optical frequencies over fiber networks represents a transformative capability at the forefront of precision metrology^1–3^, forming the critical infrastructure for a new era of scientific exploration. This technological advancement enables a new generation of groundbreaking experiments, including intercontinental optical clock comparisons^4–7^, very-long-baseline interferometry^8,9^, next-generation geodesy^10,11^, and rigorous tests of fundamental physics^12–14^.

Central to this advancement is the optical phase-locked loop (OPLL), which actively suppresses the pervasive phase noise induced by transmission fibers. Through continuous refinement, OPLL-based optical frequency dissemination (OFD) systems have achieved remarkable fractional frequency instabilities at the ${10}^{-18}$ to ${10}^{-20}$ level over intracontinental distances^4,15,16^, marking a significant milestone in frequency metrology. However, performance remains far from reaching the fundamental limit due to residual noise from imperfect cancellation and the frequent loss of lock.

First, the OPLL operates under the assumption that round-trip phase noise is symmetric, enabling noise cancellation by compensating for half of the total round-trip phase noise—this yields zero deviation when the forward (${v}_{f}$) and the backward (${v}_{b}$) optical signals share the same frequency^17^. In practice, however, fiber links are subject to significant parasitic reflections. All long-distance optical frequency transfer employs frequency shifting at the remote end to tag the signal, exploiting the resulting frequency difference ($\Delta \nu$) to suppress interference from single-pass parasitic reflections^18,19^. Nevertheless, $\Delta \nu$ disrupts the symmetry required for ideal noise cancellation in the OPLL, leading to a residual noise on the order of ${10}^{-7}$, i.e., $\left(1/{v}_{f}-1/{v}_{b}\right)/2\approx \Delta \nu /2{v}_{0}$. Importantly, as long as the phase compensation value is recorded, this deviation can be calculated and corrected.

On the other hand, OPLLs frequently lose lock under high-noise conditions^5,15,16,20^, and although anomalies can be removed afterward, this still degrades link stability. To date, only one low-noise OFD link exceeding 1000 km has been reported to operate continuously for several days without losing lock^21^. The OPLL is a typical negative feedback system, and the loss of lock typically occurs when excessive system noise causes the feedback error signal to exceed the monotonic dynamic range, rendering the feedback ineffective. OPLL robustness can therefore be enhanced by expanding the monotonic dynamic range and/or reducing the noise level.

Conventional phase discriminators, such as RF mixers, rely on zero-phase cross-correlation between reference and detected heterodyne signals and are constrained to a monotonic dynamic range of $\pi$ radians (half cycle)^17^. Current approaches to extend this range include frequency division of the optical beat note prior to phase discrimination^4,5,15,22^ and the use of digital phase-unwrapping algorithms^23^. Nevertheless, to date, the maximum achieved monotonic dynamic range remains below 1000$\pi$ radians of optical phase^22^, which is insufficient for many applications. Although digital unwrapping algorithms can theoretically extend the dynamic range indefinitely^23^, they often fail under fast phase transient conditions due to error propagation and limited sampling rates. Enhancing the OPLL’s monotonic dynamic range alone cannot fully prevent frequent lock loss.

Cascading OFD links is an effective method for extending link length and reducing OPLL noise levels. However, in very long cascaded configurations, accumulated noise across multiple stages can still compromise system robustness. This issue is further exacerbated by the fact that each OFD link typically has a similar span^22^, causing its OPLL gain peaks to align at approximately the same frequency, thereby amplifying the overall noise accumulation. While noise suppression techniques based on ultra-stable laser tracking have been proposed and experimentally demonstrated^16^, the practical effectiveness of parallelization strategies over long-distance links has not yet been experimentally validated.

In this work, we present a scalable OFD system that not only corrects bias in fiber noise compensation but also improves robustness by extending the monotonic dynamic range of the OPLL and suppressing optical phase noise. We achieve high-resolution optical phase recording with sub-radian precision over a theoretically unlimited range by directly measuring the phase of the radio frequency (RF) optical beat note through precise time-of-flight measurements of digitally conditioned RF signals. Since noise is detected in the digital domain, we enable real-time link noise compensation using bias-corrected phase information. Furthermore, we implement a hertz-level optical bandpass filter to mitigate accumulated phase noise in long and noisy cascaded links. To validate the proposed techniques, we deployed the developed system in a field experiment to establish a 2067-km round-trip cascaded OFD link. The fiber link exhibits a high noise density of approximately 5000 ${\mathrm{rad}}^{2}\,{\mathrm{Hz}}^{-1}\,{\mathrm{km}}^{-1}$ at 1 Hz. Nevertheless, the system achieves a fractional frequency instability of $2.9\times {10}^{-21}$ at 1 day, incorporating an optical phase noise filter at the 1267-km node. Notably, the phase noise density of the full 2067-km link is lower than that of the 1267-km segment, demonstrating that the link can be extended to arbitrary lengths when equipped with intermediate optical filtering stages.

## Results

### Development of ultra-stable long-haul OFD links

Building upon the previously introduced conceptual framework, we have developed a long-distance OFD system centered on relay station units, capable of real-time, bias-free noise compensation and robust operation in high-noise field-deployed fiber environments. The system comprises three components: relay stations, bidirectional optical amplifiers, and ultra-stable lasers with 1555.12 nm wavelength and sub-Hertz linewidth. Relay stations are equipped with real-time fiber noise suppression functionality, support cascaded deployment, and feature dedicated ports for external reference signal input. The ultra-stable laser provides a highly coherent optical signal at the designated frequency. By employing a loosely phase-locked control scheme, the relay stations thus actively suppress phase noise from the incoming carrier laser, effectively mitigating accumulated phase disturbances that would otherwise lead to OPLL unlocking over extended distances. Furthermore, both integrated and external bidirectional optical amplifiers within the relay stations enhance the signal-to-noise ratio (SNR) of the detected optical carrier, compensating for attenuation-induced degradation due to fiber transmission loss. Relay stations and bidirectional optical amplifiers support remote parameter configuration and data acquisition.

To validate the performance of the OFD system, we conducted field tests over a 2067-km round-trip optical fiber link connecting Shanghai to Taihu County, Anqing City, Anhui Province, employing our self-developed equipment. Key performance metrics, including link noise and system stability, were accurately calibrated.

### Relay station for scalable OFD

**Fig. 1:**
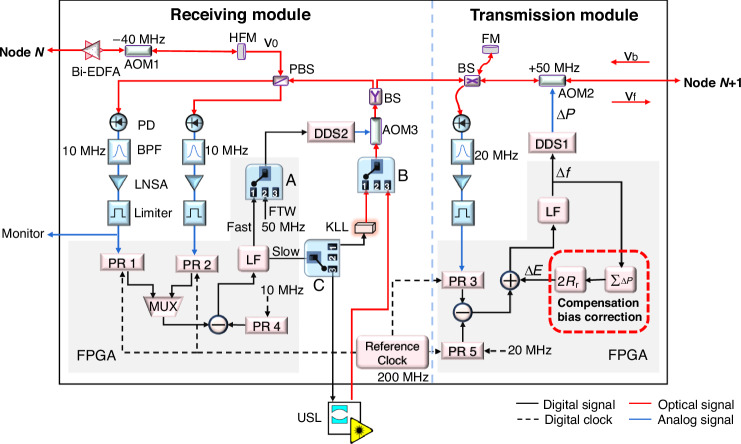
Schematic of the relay station. The relay station comprises receiving and transmitting modules, enabling integration with various external devices and supporting flexible configuration of multiple operational modes. All digital circuit functionalities are realized using a single field-programmable gate array (FPGA). HFM half-pass Faraday mirror, PBS polarizing beam splitter, BS beam splitter, FM Faraday mirror, PD photodetector, MUX multiplexer, AOM acousto-optic modulator, USL ultra-stable laser, KLL kilohertz-linewidth laser. Electronic modules include BPF band-pass filter, LNSA low-noise self-adaptive-gain amplifier, PR phase recorder, LF loop filter of OPLL, DDS direct digital synthesizer

The relay station (Fig. 1) serves as the central component of our scalable OFD architecture, capable of flexibly switching among multiple operational modes through switch selection and parameter configuration. The mapping between switch states and corresponding operating modes is shown in Table 1.

**Table 1:** Settings of relay station modes

Switch A	Switch B	Switch C	Relay station mode
2	3	2	Initial node
1	1	1	Cascaded node
1	3	3	Cascaded noise purification node

The processing flow and control logic for the laser signal in the relay station differ across various operational modes. In the initial node mode, an external reference laser is injected into the relay station via a dedicated interface and directly routed to the transmission module through an acousto-optic modulator (AOM3). In this configuration, the direct digital synthesizer (DDS2) driving AOM3 is set to operate in fixed-frequency output mode. In the cascaded mode, a “dual-dimensional control” strategy is implemented to enable cascaded locking and regeneration of the laser signal. Here, the local reference laser, with a linewidth on the kilohertz scale, is phase-locked to the incoming signal from the upstream station using an OPLL. During the locking process, coarse frequency adjustments over a wide range are performed via the laser’s built-in piezoelectric transducer (PZT), while fine, broadband tuning at the kilohertz level is achieved through DDS2. Once phase lock is established and the regenerated signal reaches stable operation, it is delivered to the transmission module. In the cascaded purification mode, the primary objective shifts to signal purification. To significantly improve frequency stability, the local laser source is switched to an ultra-stable laser with a sub-hertz linewidth. The OPLL-based phase-locking mechanism remains in use, but the control parameters are tailored to the characteristics of the ultra-stable laser: DDS2 performs loose phase locking within a hertz-level bandwidth, while large-range frequency adjustments are executed through the laser’s native digital frequency modulation interface to maintain long-term lock integrity. The purified signal is then fed into the transmission module, enabling the cascaded distribution of low-phase-noise optical signals.

The transmission module is specifically designed for fiber noise compensation, with a Michelson interferometer serving as its core component. This interferometer features asymmetric arm lengths and polarization insensitivity, enabling high-precision measurement of the additional phase noise accumulated by the optical signal during round-trip propagation through the fiber. Structurally, the short arm terminates at a Faraday mirror (FM), which provides a stable phase reference for noise detection, while the long arm acts as a signal transmission channel, delivering the optical signal to the next relay station. Upon arrival at the subsequent station, the signal undergoes frequency shifting via AOM1 before being reflected by a half-pass Faraday mirror (HFM), thus completing the round-trip path. The fiber-induced noise is embedded within the heterodyne RF signal captured by the photodetector integrated in the interferometer. This signal is processed by a field-programmable gate array (FPGA)-based phase recorder that exhibits a wide and monotonic dynamic range in the OPLL, thereby facilitating the calibration of noise compensation deviations. Phase noise compensation is achieved by adjusting the output phase of DDS1—the driver of acousto-optic modulator AOM2—thereby actively suppressing the measured phase noise. A dedicated FPGA-based logic module is incorporated into the system to perform real-time computation and feedback control, ensuring high-precision phase compensation.

The receiving module is designed to regenerate the optical carrier signal transmitted from the preceding station, accomplished by employing an OPLL to lock the local laser to the incoming signal. The control reference signal, analogous to that in the transmission module, is acquired via RF phase recorders from the optical heterodyne interference signal, with the control scheme implemented as previously described. A critical challenge arises from polarization state fluctuations in the out-of-loop RF signal used for laser locking, which can severely degrade the SNR of the beat note and subsequently lead to OPLL unlock events. To mitigate this issue, a polarization beam splitter (PBS)-based heterodyne interferometer is deployed at each receiving module^24^. This subsystem enables real-time monitoring of orthogonal polarization components and dynamically selects the channel with relatively high SNR, thereby ensuring robust OPLL operation.

Within the relay station, all frequency signal generation and measurement operations are referenced to a common local RF oscillator. Furthermore, the sum of all optical frequency shift components at the station is constrained to zero (i.e., ${v}_{0}$ is identical in each node), thereby relaxing the stability and accuracy requirements on the local RF reference^22^.

### Bias-free noise compensation

To achieve accurate noise compensation, the OPLL incorporates two key features: (1) the integration of digital phase noise measurement (the phase recorder) and phase compensation (the DDS), which enables precise determination of the required compensation value to support bias calibration; and (2) a dedicated digital compensation calibration functional module(Located within the red dashed box in Fig. 1).

RF phase recorders based on time-to-digital converters (TDC)^25^ are employed to measure the fiber-induced phase noise. The timing reference is derived from the common frequency reference (200 MHz). As illustrated in Fig. 1, a high-speed photodetector first converts the optical heterodyne beat note into an electrical signal, which is then passed through a band-pass filter (20 MHz) to suppress out-of-band noise components. Subsequently, a low-noise, adaptive-gain amplifier stabilizes the signal amplitude to ensure sufficient drive capability for downstream digital logic circuits, facilitating the generation of a clean square wave. This step effectively minimizes the influence of digital quantization noise and maintains high fidelity in the digital signal domain. Then, a coarse time interval is generated by aligning the rising edge of the input signal with the nearest edge of the reference clock. Subsequently, this signal pair is fed into the TDC for high-precision time interpolation using an FPGA-based time chain architecture, achieving sub-nanosecond resolution^25,26^, which corresponds to sub-femtosecond precision at optical frequencies. When the resolution of the signal phase measurement is much smaller than the period of the signal, the accuracy of the period during phase measurement can be guaranteed. In the system, the beat frequency signal is 20 MHz, that is, the period time is 50 ns, corresponding to approximately 5 fs of optical phase change. When the resolution of the TDC measurement is 1 ns, the single optical phase measurement resolution is 0.1 fs. The final measured time value, ${T}_{{\rm{m}}}$, is obtained by combining the coarse time with the fine time interpolation result. Concurrently, the same time measurement process is applied within the FPGA to the internal reference frequency signal to yield the reference time ${T}_{r}$. Finally, the optical phase error is calculated as $\Delta \varphi =2\pi \times \left({T}_{{\rm{m}}}-{T}_{{\rm{r}}}\right)/{T}_{0}$, where ${T}_{0}$ denotes the period of the heterodyne signal, approximately 50 ns. Note that the high-rate digital phase recording eliminates cycle ambiguity entirely, thereby enabling robust operation of the OPLL even under extremely noisy conditions.

As previously mentioned, the noise compensation deviation is determined by the round-trip optical frequency difference (80.0 MHz) and the optical carrier frequency (193.4 THz), yielding a deviation coefficient ${R}_{r}=2.07\times {10}^{-7}$. Since all signals involved are digital, the corresponding compensation term can be efficiently computed and applied in real time. The input to the bias compensation module is the phase correction quantity $\Delta P$ generated by the OPLL loop filter to drive the DDS, which is calculated as the product of the DDS frequency increment $\Delta f$ and the control cycle duration $T$ (100 $\mu s$). Subsequently, the accumulated phase compensation $\sum \Delta P$ used for link noise suppression is multiplied by twice the residual coefficient ${R}_{r}$ to produce the error offset $\Delta E$, given by $\Delta E=2{R}_{r}\sum \Delta P$. This offset is then fed back into the subsequent control stage (Fig. 1), thereby completing the correction of the bias noise compensation arising from the bidirectional laser frequency difference. The performance improvement of bias-free compensation technology can be obtained by comparing and analyzing the link instability results with and without the compensation function enabled. The specific results are presented in the “Relative instability of the OFD link” section.

### Construct a 2067-km field OFD link

**Fig. 2:**
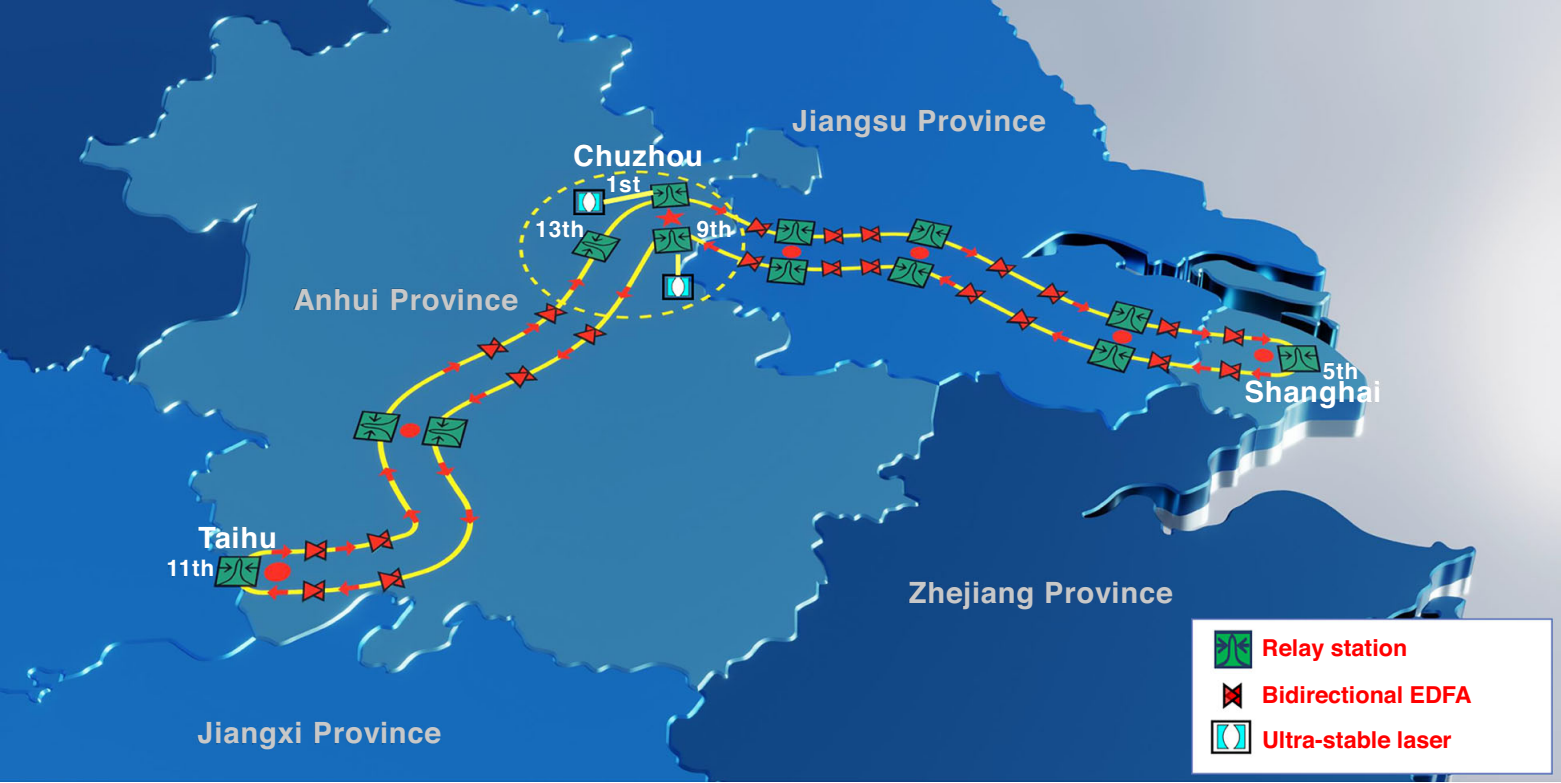
The geographical location and device distribution of the 2067-km OFD link. The system divides the link into 12 segments using 13 cascaded relay stations, with each segment ranging from 200 to 300 km. Thirty-four bidirectional optical amplifiers are deployed at independent small nodes along the link. The optical fibers consist of paired strands within the same cable duct provided by a telecommunications operator, spanning 18 metropolitan areas across East China from Shanghai to Anhui Province

As shown in Fig. 2 Chuzhou was selected as both the starting and terminating point of the link due to its favorable deployment conditions. To mitigate the impact of high fiber noise, a cascaded architecture is implemented to reduce the phase noise burden on the OPLLs. The link is segmented into 12 segments using 13 relay stations. The frequency transfer signal is generated by a free-running ultra-stable laser and first propagates eastbound to Shanghai before returning to Chuzhou station, covering a round-trip distance of approximately 1267 km. At the 9th node (Chuzhou), a second free-running ultra-stable laser is integrated to form a cascaded noise purification stage. Subsequently, the signal proceeds westbound to Taihu station and returns. Since both ends of the link are located at Chuzhou, the output signal is looped back to the origin via a short patch fiber, where it is received and monitored for end-to-end stability assessment.

The total link loss is approximately 600 dB (average attenuation: 0.29 $\mathrm{dB}$ ${\mathrm{km}}^{-1}$), compensated by 34 remotely controlled bidirectional erbium-doped fiber amplifiers (EDFAs), each providing a gain of 1030 dB. Notably, the 9th node can be flexibly switched between standard cascading mode and cascaded noise purification mode, allowing direct evaluation of the performance of the noise purification unit.

### The distance scalability of high-noise OFD links

**Fig. 3:**
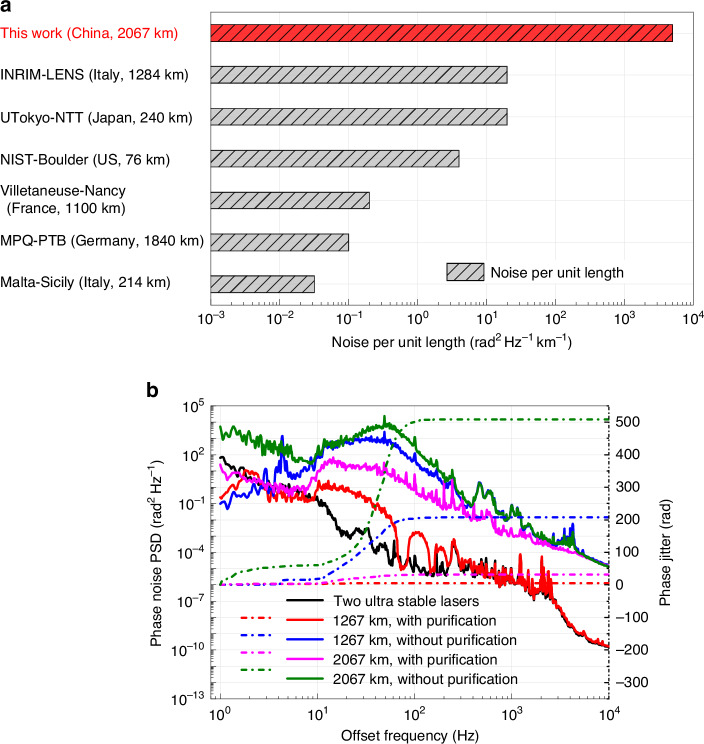
Phase noise of OFD links. **a** Fiber noise level (at 1 Hz) comparison of OFD links. Malta-Sicily: Italy, submarine fiber links, 214 km^27^; MPQ-PTB: Germany, 1840 km^15^; Villetaneuse-Nancy: France, 1100 km^5^; NIST-Boulder: US, 76 km^28^; UTokyo-NTT: Japan, 240 km^20^; INRIM-LENS: Italy, 1284 km^16^. **b** Phase noise power spectral density (PSD) and phase jitter. Phase noise PSD of the beat note between two independent ultra-stable lasers at Chuzhou(black). Noise for the 1267 km link with (red) and without (blue) noise purification. Noise for the full 2067 km link with (magenta) and without (green) purification. The left vertical axis corresponds to PSD (solid lines), and the right one to phase jitter (dashed lines)

The environmental noise profile encountered along our field-deployed fiber link presents unprecedented challenges for stable frequency dissemination. Urban segments comprising 9.2 km of aerial fiber infrastructure experience intense traffic-induced vibrations and thermal fluctuations, generating exceptional phase noise levels of 5000 ${\mathrm{rad}}^{2}\,{\mathrm{Hz}}^{-1}\,{\mathrm{km}}^{-1}$ at 1 Hz (Fig. 3a)—values exceeding those reported for dedicated fiber networks by orders of magnitude^4,5,15,16,20,21,27,28^.

Transient environmental disturbances can cause extreme optical phase changes exceeding 10,000 *π* radians within a 1-ms time interval—a situation that typically leads to catastrophic failures in traditional analog OPLLs. Our direct RF phase detection design greatly overcomes these limitations by offering a theoretically unlimited phase tracking range and an amplitude dynamic range > 60 dB, ensuring long-term lock retention even under these extreme noise conditions.

The phase noise spectra of the OFD link are shown in Fig. 3b. While cascaded noise suppression achieves substantial stabilization, noise continues to accumulate exponentially over extended distances. The servo-induced resonance between 10 Hz and 5 kHz significantly amplifies phase noise, clearly visible especially in configurations without a noise purification node (blue and green solid curves in Fig. 3b). Upon activation of the noise purification node deployed at the 1267-km mark, laser phase noise above tens of hertz is suppressed to a level comparable to that at the originating node (red solid curve). Notably, after full transmission over 2067 km, the final output exhibits superior noise characteristics relative to the intermediate 1267-km segment (magenta solid curve). Comprehensive phase noise analysis confirms a 20 dB reduction (green and magenta solid curves in Fig. 3b), phase jitter by more than an order of magnitude (green and magenta short dash-dotted curves in Fig. 3b). These results demonstrate that incorporating a single purification node can extend the OFD link by at least 800 km, with potential for further extension limited only by practical deployment constraints rather than fundamental physical barriers.

In principle, the length of OFD links can be extended without a fundamental limit by incorporating purification nodes. This is achievable because ultra-stable frequency signals are transmitted here—during the transmission of such signals, only low-frequency noise needs to be preserved to reconstruct the signal at the remote end, while link noise with higher frequencies can be effectively filtered out. To clarify this point, the purification mechanism, transfer function, and simulation verification results are elaborated in the supplementary materials, confirming that intermediate purification can effectively extend the transmission distance of links. It should be noted that noise within the low-frequency band accumulates to a certain extent, thereby degrading the stability of the links, but this can be further suppressed by adopting technical means such as better ultra-stable lasers and narrower filtering bandwidths.

### Relative instability of the OFD link

**Fig. 4:**
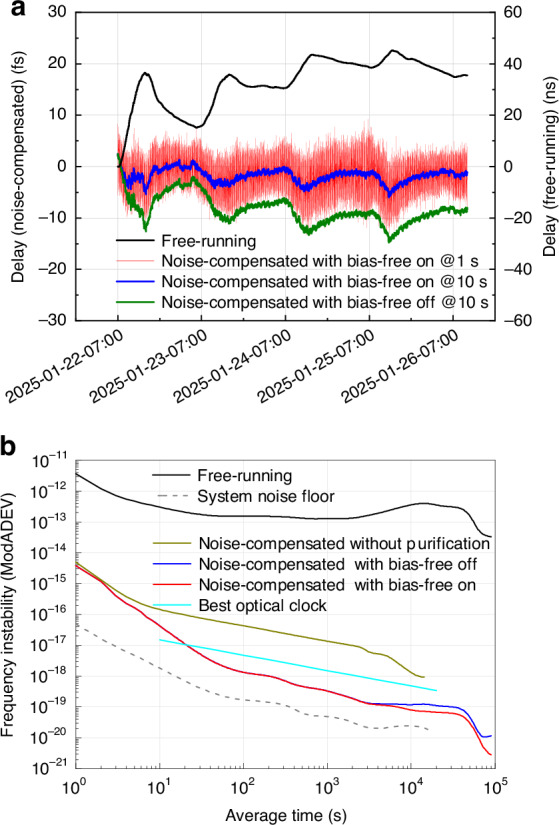
Performance of the 2067-km OFD link. **a** Time equivalent of optical carrier phase change in the link: free-running OFD link (black); noise-compensated OFD link with bias-free on (red/blue) and off (green). Long-term residual drift correlates strongly with temperature cycles. **b** Fractional frequency instability: free-running (black); noise-compensated OFD link with bias-free on $2.9\times {10}^{-21}$ at 1 day (red) and off $0.9\times {10}^{-20}$ at 1 day (blue). System noise floor is the instability of relay stations (gray dot), measured under short fiber connection conditions

By comparing the signal phases at both ends of the OFD link, we characterized the system’s time drift and frequency instability. The evolution of propagation delay highlights the exceptional noise suppression capability of our architecture (Fig. 4a). The uncompensated link exhibits a pronounced diurnal fluctuation with a peak-to-peak amplitude of approximately 55 ns—recorded via the OPLL compensation signal—that closely follows environmental temperature cycles. Our bias-free compensation scheme reduces the residual time drift to ~6 fs, clearly surpassing the theoretical residual limit of 11 fs ($2\times {10}^{-7}\times 55$ ns) inherent in conventional noise compensation methods. Data analysis further confirms that disabling bias correction leads to a degradation of time drift to ~15 fs, consistent with theoretical expectations. The remaining ~6 fs residual is primarily attributed to polarization-mode dispersion (PMD) in field-deployed fibers, which represents a fundamental limitation for current OFD systems and will be the focus of our next research phase.

The fractional frequency instability, evaluated using the modified Allan deviation, demonstrates record-breaking performance (Fig. 4b). At an averaging time of one day (*τ* = 1day), the instability reaches $2.9\times {10}^{-21}$—threefold lower than that achieved with conventional bias compensation techniques.

**Fig. 5:**
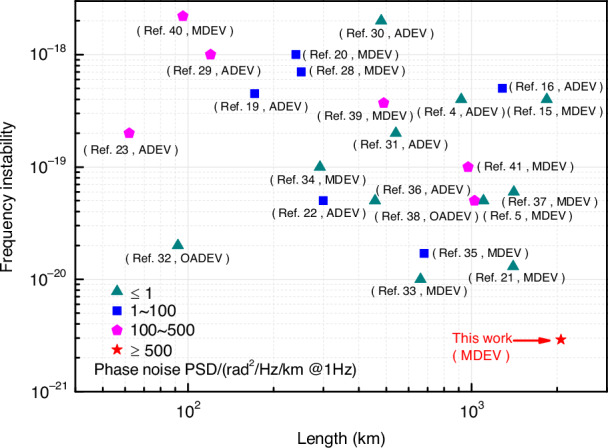
Performance comparison of long-haul OFD systems: length, frequency instability, and fiber noise level. Each point represents a reported OFD link: distance on the horizontal axis, instability on the vertical, and point style indicating noise level. Data are from Refs. ^4,5,15,16,19–23,28,36–48^ (only selected representative works are shown for clarity). This work demonstrates the longest and most stable OFD link under high fiber noise conditions. MDEV modified allan deviation, ADEV allan deviation

Thanks to the integration of noise purification, direct RF phase detection, and real-time adaptive correction, our system maintains continuous lock even under long-haul, high-noise conditions (phase noise > 5000 ${\mathrm{rad}}^{2}\,{\mathrm{Hz}}^{-1}\,{\mathrm{km}}^{-1}$ at 1 Hz). This robustness provides a critical technical foundation for achieving ultra-high stability. A comprehensive comparison with state-of-the-art long-haul OFD systems (Fig. 5) confirms that our approach outperforms all prior demonstrations in both transmission distance and fractional frequency instability, achieving unprecedented precision in optical frequency.

## Discussion

We have developed bias-free, cascaded relay stations for OFD links, effectively eliminating the detrimental impact of residual compensation errors historically limiting link stability. Furthermore, the integrated application of direct optical phase recording via RF extrapolation, noise purification, and real-time adaptive correction ensures exceptional operational reliability of the OFD system. In experimental demonstrations over a 2067-km fiber link, we achieved a fractional frequency instability of $2.9\times {10}^{-21}$ at an averaging time of one day, while operating under extreme phase noise conditions exceeding 5000 ${\mathrm{rad}}^{2}\,{\mathrm{Hz}}^{-1}\,{\mathrm{km}}^{-1}$ at 1 Hz—surpassing the theoretical performance limits of conventional biased noise compensation schemes. Critically, our approach does not rely on specialized low-noise fibers but instead leverages existing telecommunications infrastructure, a key advantage that significantly expands its feasibility for widespread deployment and diverse scientific and technological applications. The specific links also need to consider complexity, cost, and implementation conditions; in some special cases, such as the frequency transmission of submarine optical cables, more design restrictions may be encountered. We hope that a more systematic solution can be formed in the future.

This advancement addresses the pressing needs of next-generation global precision applications, such as the redefinition of the SI second via optical clock networks^1,2,4–7^, high-precision relativistic geodesy and seismic sensing^10,11,29,30^, distributed quantum information networks^31,32^, enhanced dark-matter detection strategies^33,34^, and revolutionary experiments in fundamental metrology^1,2,35^. By bridging the critical gap between laboratory-level stability and practical field deployment, our work establishes the foundation for an intracontinental-scale precision metrology infrastructure that will simultaneously accelerate scientific discovery and drive technological innovation across multiple domains.

## Materials and methods

### System integration

The experimental implementation of the cascaded fiber noise suppression system is organized into two modular, rack-mounted subsystems: a thermally stabilized optical assembly (1U height) and an electronic control unit (3U height). The optical module integrates all critical passive components—excluding the AOM and laser sources—within a compact, shielded enclosure (size sub-$10\times 15\times 5$ cm), providing milli-Kelvin thermal stability to minimize thermo-optic phase drifts in the fiber paths.

A comprehensive digital signal processing chain is implemented on a Xilinx Spartan-6 FPGA, performing real-time fiber noise cancellation, regenerative heterodyne phase stabilization, and advanced phase-domain control. Key functionalities include event-timing-based phase recording, adaptive loop filtering, and correction of phase compensation bias induced by bidirectional propagation asymmetry. Theoretically, the data throughput and storage requirements for continuous TDC phase recording are relatively large, which may limit the ultra-long-term operation of the system. Instead of directly storing the data from the phase recorders, we store and calculate the difference between the two phase recorders and use the method of cumulative summation of data to replace the storage of each data point, thereby significantly reducing the demand for data throughput and storage. Currently, each relay device’s FPGA uses about 2000 slice registers, accounting for less than 20% of the total resources. This monolithic integration on a single chip enhances system reconfigurability, reduces footprint, and improves reliability.

Deployed within standard telecommunications facilities, each noise suppression node operates alongside bidirectional EDFAs and ultra-narrow linewidth lasers. The system incorporates 4G-LTE cellular backhaul connectivity for cloud-based remote management, enabling four key capabilities: (1) software-defined parametric optimization of stabilization loops; (2) real-time telemetry collection for noise suppression performance and laser lock metrics; (3) comprehensive remote configuration, diagnostics, and operational analytics; (4) self-recovery functionality after interruption or loss of lock. This design meets carrier-class requirements for modularity, serviceability, and reliability, ensuring seamless integration into existing optical network infrastructure while maintaining ${10}^{-21}$ level frequency instability.

## Supplementary information

Supplementary Information for: 10–21-Level optical frequency dissemination over 2067 km of noise-loaded field-deployed fiber network

## Data Availability

All data supporting the main findings of this work are available within the paper, or available from the corresponding author upon reasonable request.
