# Supplementary material for: 10−21-Level optical frequency dissemination over 2067 km of noise-loaded field-deployed fiber network
Source: Light Sci Appl. 2026 Jun 22;15:276. doi: 10.1038/s41377-026-02299-1 (PMC13287666; doi:10.1038/s41377-026-02299-1)
Supplement: Supplementary file 1 — Supplementary Information for: 10–21-Level optical frequency dissemination over 2067 km of noise-loaded field-deployed fiber network [file 41377_2026_2299_MOESM1_ESM.docx]

**Supplementary Information for: 10^-21^-Level optical frequency dissemination over 2067 km of noise-loaded field-deployed fiber network**

**Fa-Xi Chen**^1,2^**†, Li-Bo Li**^2^**†, Jiu-Peng Chen**^1,2^**†, Kan Zhao**^2^**, Jian-Yu Guan**^1^**, Yang Xu**^1,3^**, Lei Hou**^1^**, Fei Zhou**^1,2^**, Cheng-Zhi Peng**^1,3^**, Qiang Zhang**^1,2,3^***, Hai-Feng Jiang**^1,3^***, and Jian-Wei Pan**^1,3^*

1 Hefei National Laboratory, University of Science and Technology of China, Hefei 230088, China.

2 Jinan Institute of Quantum Technology and CAS Center for Excellence in Quantum Information and Quantum Physics, University of Science and Technology of China, Jinan 250101, China.

3 Hefei National Research Center for Physical Sciences at the Microscale and School of Physical Sciences, University of Science and Technology of China, Hefei 230026, China.

| Names | Email addresses | Telephone number |
| --- | --- | --- |
| Fa-Xi Chen | chenfaxi@jiqt.org |  |
| Li-Bo Li | lilibo@jiqt.org |  |
| Jiu-Peng Chen | chenjiupeng@jiqt.org |  |
| Kan Zhao | zhaokan@jiqt.org |  |
| Jian-Yu Guan | jyguan@hfnl.cn |  |
| Yang Xu | xuyang2013@ustc.edu.cn |  |
| Lei Hou | lhou@ustc.edu.cn |  |
| Fei Zhou | zhoufei@jiqt.org |  |
| Cheng-Zhi Peng | pcz@ustc.edu.cn |  |
| Qiang Zhang | qiangzh@ustc.edu.cn |  |
| Hai-Feng Jiang | hjiang1@ustc.edu.cn | +86 18591945105 |
| Jian-Wei Pan | jwpan-office@ustc.edu.cn |  |

## Detailed overview of the cascaded long-haul OFD link

The ultra-stable frequency dissemination infrastructure is implemented over a 2067 km fiber loop connecting two parallel telecommunication fibers within a shared cable duct, establishing a link between the western (Taihu) and eastern (Shanghai) terminals. The path traverses 18 major metropolitan areas across China. Twelve actively stabilized repeater stations subdivide the entire link into segments (each < 230 km) to ensure robust phase noise suppression. The system employs dense wavelength division multiplexing (DWDM) to enable simultaneous ultra-precision frequency dissemination and conventional data traffic over the same fiber infrastructure.

The optical frequency reference signal is generated at the Chuzhou Station and propagates bidirectionally through the looped network via Shanghai and Taihu before returning to Chuzhou for comprehensive stability assessment. The total link loss, incorporating fiber attenuation, splice points, and connector losses, amounts to approximately 600 dB, corresponding to an average attenuation of 0.29 $dB {km}^{-1}$. To compensate for this substantial loss, 34 remotely controllable bidirectional erbium-doped fiber amplifiers (bi-EDFAs) are deployed across the 18 metropolitan nodes (see Tab. [2](#bookmark37)). Each bi-EDFA provides 10–30 dB of bidirectional gain.

Critical measures were implemented to ensure transmission integrity and amplifier stability. Dual-pumping schemes are employed in all amplifiers to mitigate the risk of self-oscillation under high-gain conditions. Fusion splicing losses are rigorously controlled to below 0.05 dB per splice. Backward pumping amplification is utilized in both propagation directions at the initial stage to minimize nonlinear optical effects. Furthermore, optical filters with a 25 GHz bandwidth are installed before and after each pump stage to suppress out-of-band amplified spontaneous emission (ASE) noise, thereby ensuring amplifier stability and enhancing the overall signal-to-noise ratio.

**Table 1: Segment-wise attenuation characteristics of the 2067 km cascaded fiber link.** Fiber link1 and link2 denote the two parallel fibers within the shared duct.

| **Segment** | **Length** | **Attenuation of fiber link1** | **Attenuation of fiber link2** |
| --- | --- | --- | --- |
| Taihu and Qianshan | 54.2 km | 19.3 dB | 17.5 dB |
| Qianshan and Anqing | 55.5 km | 15.9 dB | 17.8 dB |
| Anqing and Tongcheng | 84.1 km | 25.5 dB | 24.7 dB |
| Tongcheng and Shucheng | 59.7 km | 18.9 dB | 20.9 dB |
| Shucheng and Hefei | 64.5 km | 21.3 dB | 19.1 dB |
| Hefei and Chuzhou | 81.8 km | 27.5 dB | 28.8 dB |
| Chuzhou and Chuzhoudong | 61.4 km | 17.5 dB | 16.4 dB |
| Chuzhoudong and Nanjing | 78.4 km | 20.6 dB | 21.3 dB |
| Nanjing and Nanjingdong | 37.0 km | 9.6 dB | 10.5 dB |
| Nanjingdong and Zhenjiang | 76.0 km | 20.8 dB | 19.9 dB |
| Zhenjiang and Danyang | 43.2 km | 11.9 dB | 13.2 dB |
| Danyang and Changzhou | 64.8 km | 16.8 dB | 18.8 dB |
| Changzhou and Wuxi | 62.7 km | 17.6 dB | 17.5 dB |
| Wuxi and Suzhou | 69.9 km | 15.7 dB | 16.9 dB |
| Suzhou and Kunshan | 51.8 km | 12.2 dB | 12.4 dB |
| Kunshan and Shanghaixi | 64.2 km | 20.3 dB | 20.3 dB |
| Shanghaixi and Shanghai | 24.5 km | 6.0 dB | 6.0 dB |

## Automatically relock function

To ensure the rapid resumption of operation of the transmission system in case of unexpected situations such as fiber breaks or light source switching, the system is designed with an automatic locking function. Each relay node continuously monitors the phase locking status. Once a loss of lock is detected, the PID feedback control is immediately turned off and the automatic locking process is initiated. This process involves scanning the frequency of the local laser — for relay nodes, this is achieved by adjusting the PZT drive voltage of the narrow-linewidth laser, while for purification nodes, it is accomplished by changing the sideband modulation frequency of the ultra-stable laser — to reposition the locking point. Subsequently, the PID locking loop is re-enabled to complete the phase recovery. Fig. S1 shows the frequency changes of the external beat frequency signal during the system's loss of lock and recovery process. In a test of a 2067-kilometer OFD system, the fiber at the first relay station was manually disconnected for about one minute and then reconnected. As a result, the first relay station was the first to regain lock, followed by the subsequent stations in sequence, and eventually the entire system resumed stable operation. The beat frequency signal shown in Fig. S1 was output from the monitoring port of this relay station. Currently, the automatic recovery time for a single relay node is less than 30 seconds, and the recovery time for the entire system depends on the number of nodes that lost lock, typically not exceeding 5 minutes. All operational monitoring data are automatically uploaded to the remote monitoring platform via the communication system, enabling centralized state monitoring and management of the system.


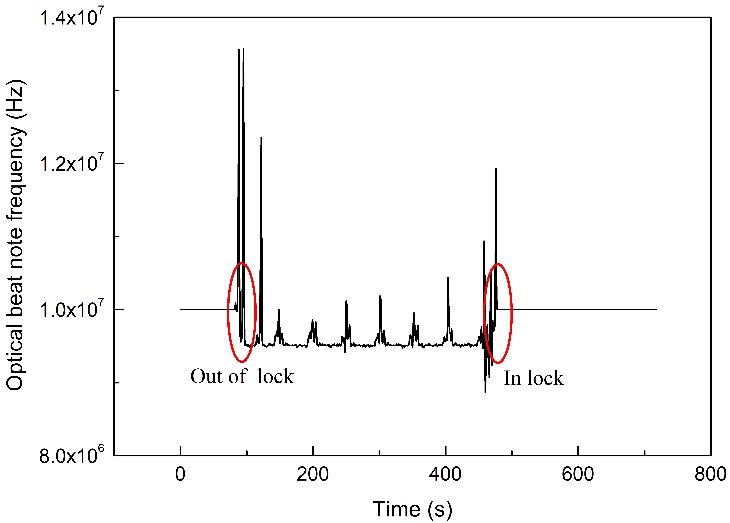


**Fig. S1****. Laser beat note frequency signal during the unlocking and automatic relocking process of the 2067-km OFD link.**

## The Allan deviation of the link

As shown in Fig. S4 of the main text, the frequency stability MDEV has improved by three times after bias correction. While using ADEV analysis, the instability has increased by nearly twice (see Fig. S2). Specifically, the noise-compensated OFD link with bias-free on achieves $3.1\times{10}^{-20}$ at 100000 s (red), while with bias-free off it achieves $5.8\times{10}^{-20}$ at 100000 s (blue) with bias-free off. This difference is attributed to the poor suppression effect of ADEV on high-frequency noise; therefore, the ADEV is higher than MDEV.


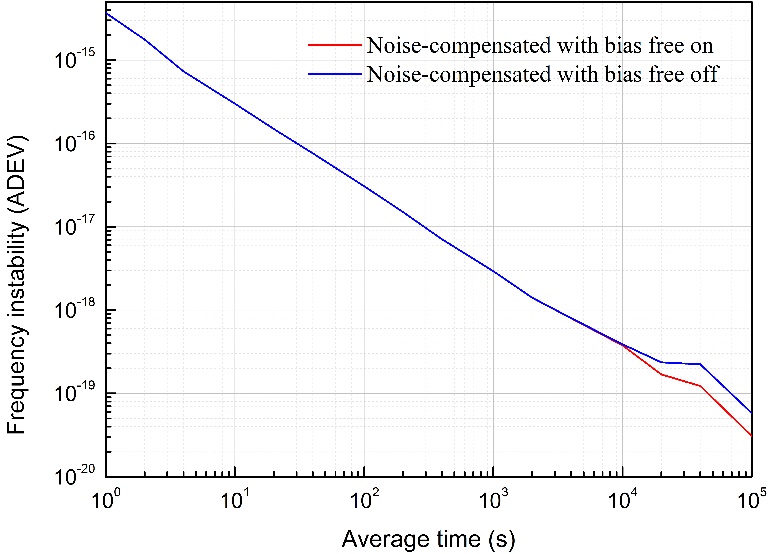


**Fig. S2. Allan Deviation (ADEV) of the 2067-km OFD link.**

## Noise purification and link extension

Fig. S3 shows the signal flow of the laser phase noise purification process. This process first measures the beat frequency signal between the laser signal to be purified and the local ultra-stable laser through a phase recorder, thereby obtaining the phase error. After being processed by the digital loop filter, this error generates a phase modulation signal, which is then loaded onto the local ultra-stable laser, and finally the purified laser signal is output. Among them, the loop filter, as the core part, is composed of a Butterworth low-pass filter and a PI controller.


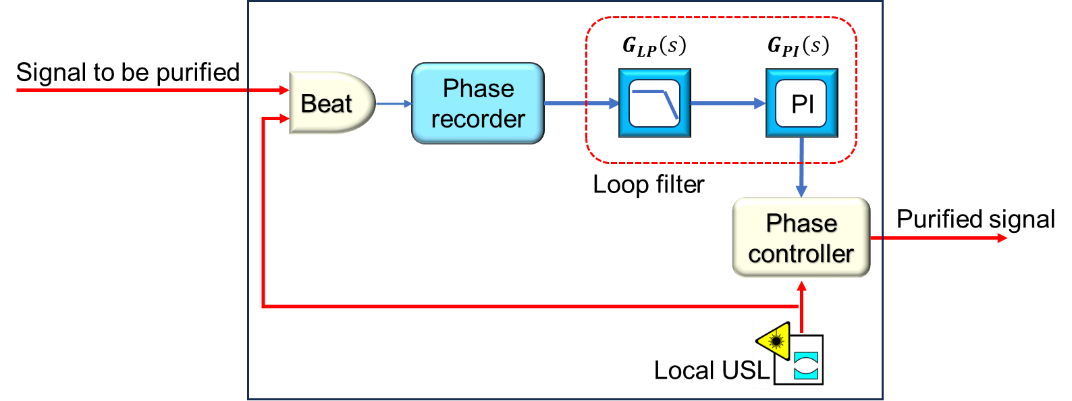


**Fig. S3.** **Schematic of purification process.**

The open-loop transfer function of the loop filter is

$$\begin{aligned} G_{open}\left( s \right)=G_{LP}\left( s \right)G_{PI}\left( s \right)=\frac{\omega_{c}^{2}}{s^{2}+\sqrt{2}\omega_{c}s+\omega_{c}^{2}}\left( K_{P}+\frac{K_{I}}{s} \right) \#\left( 1 \right) \end{aligned}$$

where $\omega_{c}=6\pi, K_{P}=1, K_{I}=5$.

The transfer function of the phase noise of the laser output after purification is

$$\begin{aligned} N_{Pur}(s)=(\frac{G_{open}\left( s \right)}{1+G_{open}\left( s \right)})N_{TBP}(s)+(\frac{1}{1+G_{open}\left( s \right)})N_{USL}(s) \#\left( 2 \right) \end{aligned}$$

where $N_{Pur}(s)$, $N_{TBP}(s)$, $N_{USL}(s)$ are the purified signal, the signal to be purified and the local ultra-stable laser respectively. The noise accumulation in the 20–200 Hz frequency band is identified as the primary cause of link unlock events. In this analysis, the phase jitter induced by noise within this band is specifically examined; the purification process achieves a suppression ratio approximately 40 dB in this frequency range.

The noise of the optical signal after 1267-km transmission (the blue solid line in Fig. S4) was taken as the signal to be purified, and the noise of the ultra-stable laser signal in the Dashu Station (the black solid line in Fig. S4) was taken as the local signal. The simulation was carried out according to the above transfer function, and the noise of the purified optical signal (the dark cyan dashed line in Fig. S4) was obtained. The measured results (the red solid line in Fig. S4) are in good agreement with the simulation results; the difference at low frequencies is due to the large environmental vibration noise in the Dashu Station, which led to additional noise in the measured purified signal.


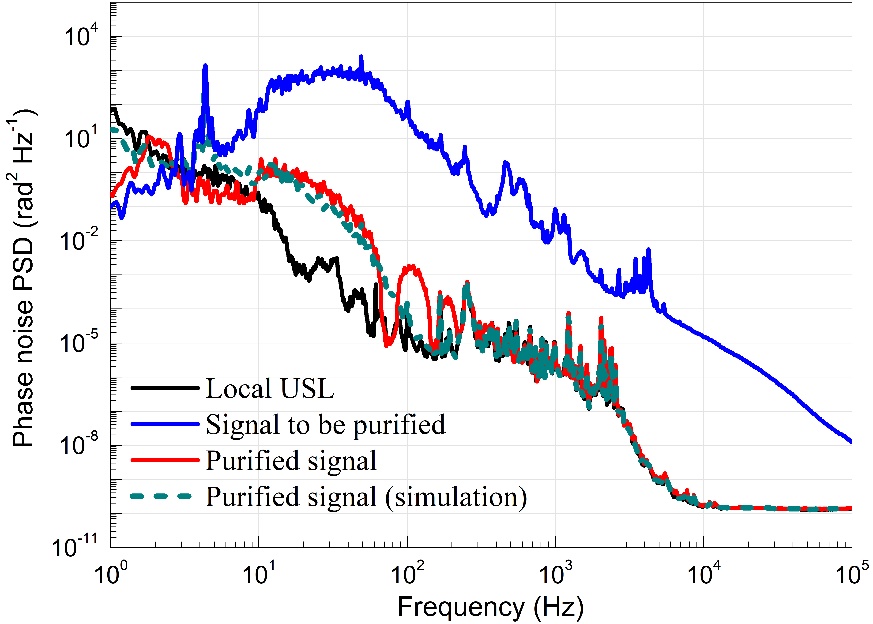


**Fig. S4:** **Noise purification results: measured and simulated laser phase noise of the 1267-km link.**

We simulate the accumulation of phase jitter of the laser signal during long-distance transmission (see Fig. S5). The initial laser signal had a phase jitter of approximately 5 rad in the 20–200 Hz frequency band. After 1267 km of transmission and 8 noise suppression loops, the jitter increased to 208 rad; when it was transmitted to 2067 km, the jitter further increased to 508 rad. This shows that the noise is amplified step by step with the transmission distance and the number of loops, and the amplification is faster with more loops. To solve this problem, we set up a purification station at 1267 km to purify the signal, which could restore the phase jitter in this frequency band to approximately 5 rad, comparable to the initial laser source, effectively blocking the step-by-step amplification of noise. After purification, the signal passed through another 800 km and four noise suppression loops, and the jitter only rose to 32 rad. Based on the above measured data, we simulated the changes in phase jitter in this frequency band under the multi-stage purification process. The results show that within 1267 km, the measured data was used, and purification was carried out approximately every 1000 km thereafter. Due to the sufficiently low loop bandwidth, the noise in this frequency band could be restored to nearly the initial state after each purification. Therefore, through periodic purification, the phase jitter can be continuously suppressed, theoretically extending the stable transmission distance of the laser signal infinitely.


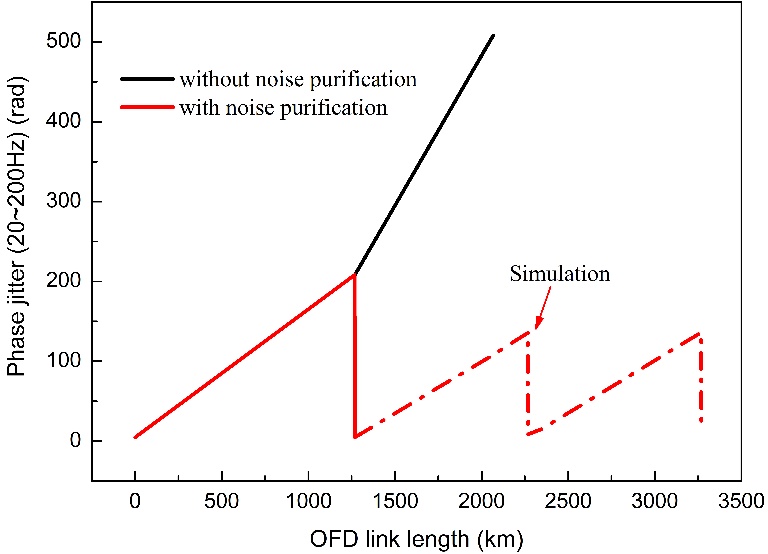


**Fig. S5: Phase jitter of a cascaded OFD link (dashed line: simulation result, solid line: measurement result).**

## Performance of international fiber links for long-haul optical frequency dissemination

**Table 2: Performance of international fiber links for long-haul optical frequency dissemination**

|  | **Location** | **Fiber type** | **Length (km)** | **Noise (rad²Hz⁻¹km⁻¹@1Hz)** | **Duration (days)** | **Cycle Slips** | **frequency instability( compensated)** |
| --- | --- | --- | --- | --- | --- | --- | --- |
| 1 | MPQ-PTB (Germany) | Dedicated fiber | 920 | 0.11 | 3 | a few per day | 5×10^-15^@1 s  <4×10^-19^@4000 s  (ADEV) |
|  |  |  | 1840 |  | 3 | 98.5% | 2.7×10^-15^@1 s  4×10^-19^@100 s  (MDEV) |
| 2 | Villetaneuse-Nancy (France) | Communication fiber | 1100 | 0.2 | 3.5 | 73 instances | 1×10^-16^@1 s  5×10^-20^@60000 s  (MDEV) |
| 3 | INRIM-LENS (Italy) | Communication fiber | 1284 | 20 | 0.23 | a few per hour | 1×10^-14^@1 s  5×10^-19^@1000 s  (ADEV) |
| 4 | UTokyo-NTT  (Japan) | Dedicated fiber | 240 | 20 | <1 | ~4×10^-3^ s^-1^ | 3×10^-16^@1 s  1×10^-18^@2600 s  (MDEV) |
| 5 | Braunschweig-Strasbourg (Germany) | Communication fiber | 1400 |  | 5.7 | 0 | 2×10^-15^@1 s  1.3×10^-20^@123000 s  (MDEV) |
| 7 | Paris-Lille (France) | Communication fiber | 680 |  | 5 | 285 data points are removed,  with an uptime of 99.93% | 4×10^-16^@1 s  1.7 ×10^-20^@65000 s  (MDEV) |
| 8 | Paris–Strasbourg (France) | Communication fiber | 1410 | <1 | 3 | uptimes 98.1%,  47% of the data have been removed | 7×10^-16^@1 s  (MDEV) |
| 10 | IQ-PTB-CORMIGK  (Germany) | Dedicated fiber | 480 | <1 |  |  | 2×10^-18^ @8200 s  (ADEV) |
| 11 | PTB-Kassel University (Germany) | Dedicated fiber | 660 | <1 | 6 | 0 | 1×10^-20^@10000 s  (MDEV) |
| 12 | LPL- LNE-SYRTE  (France) |  | 172 | <3 |  |  | 4.5×10^-19^@3200 s  (ADEV) |
| 13 | LPL-Nogent l’Artaud (France) | Communication fiber | 300 | <3 |  |  | 5×10^-20^@72000 s  (ADEV) |
| 14 | LPL-Reims  (France) | Communication fiber | 540 | 1 | <2 | below  10^−4^ s^-1^ | 2×10^-19^@30000 s  (ADEV) |
| 15 | LKB-LPL (France) | Dedicated fiber | 92 | 0.1 |  |  | 2×10^-20^@40000 s  (OADEV) |
| 16 | SYRTE-INRIM  ( France – Italy) |  | 1023 | <100 |  | 57% | 5×10^-20^@200000 s  (OADEV) |
| 17 | NMIJ-Kashiwa (Japan) | Communication fiber | 120 | 300 |  | 0 | 1×10^-18^@300 s  (ADEV) |
| 18 | SJTU-MH(China) | Communication fiber | 62 | 112 |  | 99.9% data uptime | 2×10^-19^@10000 s  (ADEV) |
| 19 | METAS-BS-ZH (Switzerland, Italy) | Communication fiber | 456 | 0.3 |  | 4.7 CS per hour. | 5×10^-20^@100000 s  (ADEV) |
| 20 | Braunschweig – LUH (UK, Germany) |  | 292 | <1 |  |  | 1×10^-19^@3200 s  (MDEV) |
| 21 | NTSC-Jinshui(China) | Communication fiber | 490 | 510 |  |  | 3.7×10^-19^@10000 s  (MDEV) |
| 22 | SIOM-SHB (China) | Communication fiber | 96 | 100 |  |  | 2×10^-18^@10000 s  (MDEV) |
| 23 | **Shanghai-Chuzhou(This work ,China)** | **Communication fiber and partly aerial optical fiber** | **2067** | **5000** | **4.1** | **0** | **3.79×10^-15^@1 s**  **2.9×10^-21^@1 day**  **(MDEV)** |
